# Drp1-mediated mitochondrial fission contributes to baicalein-induced apoptosis and autophagy in lung cancer via activation of AMPK signaling pathway

**DOI:** 10.7150/ijbs.41768

**Published:** 2020-02-21

**Authors:** Xiaohong Deng, Jingjing Liu, Lantao Liu, Xianjun Sun, Jianhua Huang, Jingcheng Dong

**Affiliations:** 1 Department of Integrative Medicine, Huashan Hospital, Fudan University, Shanghai 200040, China; 2 Department of Interventional Radiology, Putuo Hospital, Shanghai University of Traditional Chinese Medicine, Shanghai 200050, China

**Keywords:** baicalein, mitochondrial fission, apoptosis, autophagy, AMPK

## Abstract

Baicalein (BA), a natural compound extracted from *Scutellaria baicalensis* Georgi, has been reported to exert antitumor effect in various cancers. However, the underlying mechanisms have not been well demonstrated. In the present study, we focused on the relationship between mitochondrial fission and BA-induced apoptosis and autophagy. We showed that BA inhibited cell viability and induced mitochondrial apoptosis in A549 and H1299 lung cancer cells. BA induced the loss of mitochondrial membrane potential (MMP) and the release of cytochrome c and apoptosis inducing factor (Aif) from mitochondria to cytoplasm. Meanwhile, BA induced autophagy and activated autophagic flux. Furthermore, we found that BA induced mitochondrial fission and mitochondrial impairment. Blocking mitochondrial fission by mdivi-1 attenuated BA-induced apoptosis and autophagy. Moreover, BA activated AMP-activated protein kinase (AMPK) pathway. Knockdown of AMPK with lentivirus encoded AMPKα also attenuated BA-induced mitochondrial fission, apoptosis and autophagy. Our *in vivo* data confirmed that BA inhibited tumor growth and induced apoptosis and autophagy in a Lewis lung carcinoma (LLC) xenograft model via activation of AMPK/mitochondrial fission pathway. Our study highlights the critical role of AMPK/mitochondrial fission pathway in the regulation of BA-induced apoptosis and autophagy. These results revealed the molecular mechanism of the anti-lung cancer property of BA and provided novel perspectives for the application of BA in the treatment of lung cancer.

## Introduction

Lung cancer remains the first leading cause of cancer-related mortality throughout the world 1. About 1.8 million people were diagnosed with lung cancer and 1.6 million lung cancer deaths occur every year, accounting for 19% of all cancer deaths 2. Apart from the conventional treatment approaches such as surgery, chemotherapy, and radiotherapy, targeted therapy and immunotherapy for lung cancer have been developing rapidly over the years. However, these therapies work only for patients with certain biological characteristics, and they also have many limitations such as side effects and high expenses 3,4. Thus, exploring novel mechanism of known anti-tumor agents is an important way to investigate new target and molecular biological mechanism of cancer.

Increasing evidences indicate that cancer is associated with mitochondrial dynamics 5,6. Mitochondria play a fundamental role in physiological processes, ranging from cell metabolism, proliferation and differentiation to cell survival and apoptosis 7. Mitochondria exist as dynamic networks that continually change their morphology to maintain the normal shape, structure, quantity and function 8. The balance between fission and fusion dictates their morphology and distribution of mitochondrial DNA (mtDNA). Mitochondrial fission is catalyzed by dynamin-related protein 1 (Drp1). Phosphorylation at Ser616 activates Drp1 and induces mitochondrial fission 5. In contrast, Drp1 is inactivated via phosphorylation at Ser637, resulting in decreased mitochondrial fission 9,10. Meanwhile, mitochondrial fusion requires the action of mitofusin-1 (Mfn1), mitofusin-2 (Mfn2) and optic atrophy 1 (Opa1) 8.

Apoptosis and autophagy respond to similar stresses and dictate cell fate together. Both of them are related to mitochondrial fission. Mitochondrial shape dictates apoptotic susceptibility, as Drp1-dependent mitochondrial fission is an early and critical event in apoptosis. Early studies demonstrated that mitochondrial fragmentation and clustering occur just prior to cytochrome c release and coincide roughly with activation of B-cell lymphoma 2 (Bcl2) family member Bax, which is responsible for forming pores in the outer mitochondrial membrane 11,12. In addition, the process of mitochondrial fission can separate old mitochondria for degradation via autophagy/mitophagy. Apart from inducing apoptosis, increased mitochondrial fission promotes autophagy 13,14. AMP-activated protein kinase (AMPK) is the central metabolic sensor that can be activated by a large variety of mitochondrial insults 15, including mitochondrial fission. Previous studies have indicated that activation of AMPK promotes apoptosis and autophagy 16,17.

**Figure 1:**
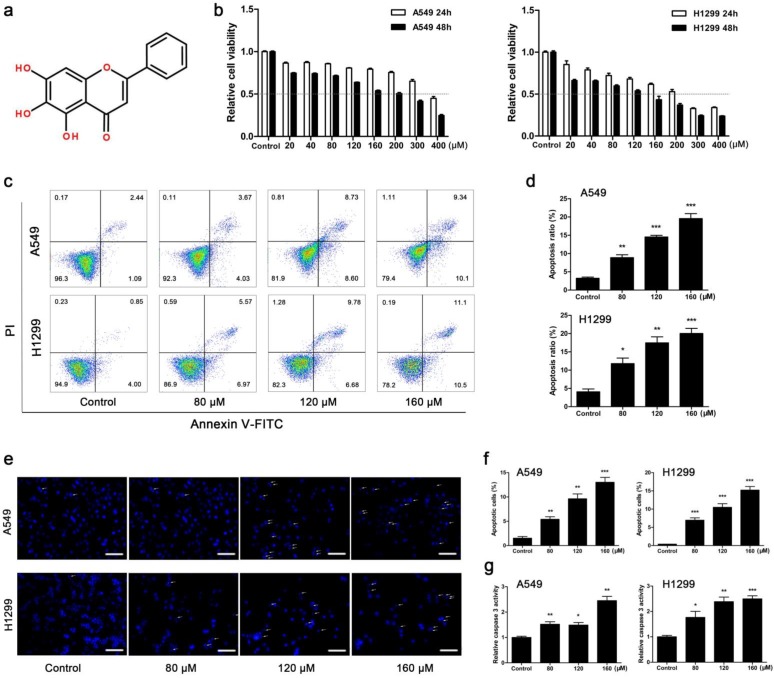
** BA inhibited viability and induced apoptosis in A549 and H1299 cells.** (a) Chemical structure of BA. (b) A549 and H1299 cells were treated with BA at concentrations of 0 ~ 400 μM. WST-1 assay was performed to examine cell viability. (c) A549 and H1299 cells were treated with BA at concentrations of 80, 120, and 160 μM. Apoptosis analyses were performed by staining with Annexin V-FITC and PI and detected using flow cytometry. (d) The ratio of apoptosis was analyzed using FlowJo. (e) Nuclear condensation and fragmentation were performed using DAPI staining and detected by fluorescent microscopy (scale bar, 100 μm). (f) Positive cell ratio was analyzed using ImageJ. (g) The activity of caspase 3 was determined with using a caspase 3 detection kit. Data were from at least three independent experiments. *p<0.05, **p<0.01, ***p<0.001

Baicalein (BA) is a principle flavonoid component mainly isolated from the roots of *Scutellaria baicalensis* Georgi which is known as Huangqin in traditional Chinese medicine. The molecular structure of BA is shown in Fig 1a. Growing evidences demonstrate BA's role in treating and preventing multiple types of cancer, including breast cancer, bladder cancer, cervical cancer, hepatocellular cancer, lung cancer, ovarian cancer, osteosarcoma, and gallbladder cancer 18-25. Inducing apoptosis 18,20,21, initiating autophagy 18, inhibiting tumor invasion and metastasis 19 and causing cell cycle arrest 26 may underlie the anticancer property of BA. However, little is known about the role of BA in mitochondrial dynamics and the relevance to BA-induced apoptosis and autophagy in lung cancer.

In the present study, we demonstrated the effects of BA on apoptosis and autophagy in A549 and H1299 lung cancer cells and a Lewis lung carcinoma (LLC) xenograft model. To explore the mechanism, we investigated the effects of BA on Drp1-mediated mitochondrial fission and AMPK signaling pathway. Our study uncovered that Drp1-mediated mitochondrial fission contributed to BA-induced apoptosis and autophagy via activation of AMPK pathway, which may provide a novel mechanistic basis for the application of BA in the treatment of lung cancer.

## Materials and Methods

### Materials and reagents

Baicalein (≥ 99%, Yousi Scientific Co., Ltd, Shanghai, China) was dissolved in DMSO at concentration of 200 mM and stored at -20 ℃. Mdivi-1, an inhibitor of Drp1, was purchased from Selleck (Huston, TX, USA). 3-Methyladenine (3-MA), an inhibitor of autophagosomes, and Bafilomycin A1 (Baf-A1), an inhibitor of H^+^-ATPase, were purchased from Selleck. Antibodies against PARP (#9542), Drp1 (#5391), AMPKα (#5831), p-AMPKα (Thr172) (#2535), LC3 (#12741), Bak (#6947) and β-actin (#3700) were obtained from Cell Signaling Technology (Boston, MA, USA). Antibodies against Caspase 3 (#19677-1-AP), Caspase 9 (#10380-1-AP), Bcl2 (#12789-1-AP), Bcl-xl (#10783-1-AP), Bax (#50599-1-AP), Cytochrome c (#10993-1-AP), Aif (#17984-1-AP), Cox IV (#11242-1-AP), Fis1 (#10956-1-AP), Opa1 (#27733-1-AP), Mfn1 (#13798-1-AP), Ndufs1 (#12444-1-AP), Sdha (#14865-1-AP), Uqcrc1 (#21705-1-AP), Atp5a1 (#14676-1-AP), p62 (#18420-1-AP), and Beclin1 (#11306-1-AP) were obtained from Proteintech (Wuhan, China). Antibody against p-Drp1 (Ser616) (#12749) was obtained from Signalway Antibody (College Park, MD, USA). Secondary goat anti-rabbit or rabbit anti-mouse antibodies were purchased from Proteintech. Fluorescent-labeled antibody Annexin V-FITC, Annexin V-APC, PI, 7-AAD and 10 × binding buffer were obtained from BD (Franklin Lakes, New Jersey, USA).

### Cell culture and treatment

Lung cancer cell lines (A549, NCI-H1299, and LLC) were obtained from Shanghai Type Culture Collection of Chinese Academy of Sciences (Shanghai, China). Cells were cultured in DMEM containing 10% (v/v) FBS and a 1% (v/v) penicillin-streptomycin solution (Gibco, Waltham, MA, USA) at 37°C in a humidified atmosphere with 5% CO_2_. The cells were treated with BA at concentrations of 80, 120, 160 μM and DMSO (control group), respectively for 48 h. Mdivi-1 (15 μM), 3-MA (5 mM) and Baf-A1 (10 nM) were applied to cells 3 h and then co-cultured with BA for 48 h.

### WST-1 cell viability

WST-1 (Beyotime, Shanghai, China) was used to assess the viability of cultured cells. Briefly, cells were seeded into a 96-well plate at a cell density of 5 × 10^3^ per well and treated with BA at indicated concentrations. Then, 10 μL of WST-1 solution was added to each well and cultured for 3 h at 37℃. The optical density (OD) absorbance was measured using a plate reader (Tecan, Männedorf, Switzerland) at 450nm.

### Cell apoptosis

Apoptosis was detected using an apoptosis kit (BD) by flow cytometry. Treated cells were collected, washed with PBS, and resuspended with 100 μL binding buffer containing 5 μL Annexin V-FITC and PI or Annexin V-APC and 7-AAD. Cell suspension was incubated for 15 min at room temperature. Apoptosis was then detected using flow cytometry (Thermo Fisher Scientific, Waltham, MA, USA) and analyzed using FlowJo V10.0.7 (BD).

### DAPI staining

Cells were seeded in a 96-well plate and treated with BA at indicated concentrations. Cultured cells were washed with PBS, fixed and permeabilized with fixative solution (Beyotime) for 20 min at room temperature. The fixed cells were incubated with DAPI solution for 10 min at room temperature. Representative images were captured using a fluorescence microscope (Zeiss, Oberkochen, Germany).

### Mitochondrial membrane potential and mitochondrial mass

Mitochondrial membrane potential (MMP) was measured using TMRE staining. Mitochondrial mass was measured using MitoTracker green FM staining. Treated cells were harvested and resuspended at 2 × 10^5^ cells/mL in DMEM containing 100 nM TMRE (Invitrogen, Waltham, MA, USA) or 200 nM MitoTracker green FM (Invitrogen). Cells were incubated for 30 min at 37℃ in the dark. MMP and mitochondrial mass were measured using flow cytometry and analyzed using FlowJo.

### Reactive oxygen species

Reactive oxygen species (ROS) was detected using a ROS assay kit (Beyotime). DCFH-DA was diluted with DMEM to a final concentration of 10 μM. Treated cells were harvested, resuspended in DCFH-DA solution at 10 × 10^5^ cells/mL, and incubated for 20 min at 37℃ in the dark. ROS was measured using flow cytometry and analyzed using FlowJo.

### Mitochondrial imaging

Cells were seeded in a 96-well plate and treated with BA at indicated concentrations. Mitochondrial red CMXRos (Invitrogen) was diluted with DMEM to a final concentration of 200 nM. Treated cells were washed with PBS and incubated with Mitochondrial red CMXRos for 30 min at 37℃ in the dark. After incubation, cells were washed with PBS and observed using a fluorescence microscope. Representative images of mitochondrial morphology were captured.

### ATP production

ATP production was measured using an ATP assay kit (Beyotime) according to the manufacturer's protocol. Briefly, treated cells in 6-well plates were lysed with 200 μL lysis buffer. Next, 20 μL lysate was added to a black-flat, clear-bottom 96-well plate containing 100μL detection solution. Chemiluminescence of the reaction mixture was measured using a plate reader.

### Mitochondrial and cytosolic fractions isolation

Mitochondrial and cytosolic fractions isolation were conducted using a cell mitochondrial isolation kit (Beyotime) according to the manufacturer's instructions. Briefly, at least 2 × 10^7^ cells were harvested, resuspended in mitochondrial isolation buffer containing 1mM PMSF, and incubated for 15 min on ice. Cell suspension was homogenized in a glass homogenizer, centrifuged at 600 × g for 10 min at 4℃. Then the supernatant was collected and centrifuged at 11000 × g for 10 min at 4℃. The supernatant stood for cytosolic fraction and the deposit was resuspended in mitochondrial lysis buffer for further assay.

### Western blotting

Cells and tissues were lysed in RIPA buffer (Thermo Fisher Scientific) containing 1% EDTA and 1 mM PMSF (Beyotime) and incubated in lysis buffer for 30 min on ice. Protein concentrations were determined using a BCA protein assay kit (Beyotime) according to the manufacturer's protocol. The whole cell lysates were subjected to 5 × loading buffer (Beyotime) and boiled for 10 min. Proteins were separated by SDS-PAGE gel electrophoresis and transferred to PVDF membrane (Millipore, Danvers, MA, USA). The membranes were blocked with 5% skim milk for 1 h at room temperature, and interacted with primary antibodies overnight at 4℃. After washed in TBS-T, the membranes were incubated with corresponding HRP-conjected secondary antibodies for 1 h at room temperature. The bands were detected using an ECL detection kit (Millipore) and a LAS-4000 mini system (Fujifilm Corporation, Tokyo, Japan) for visualization.

### Immunofluorescence staining

Cells in 96-well plates were fixed and permeabilized with Immunol staining fix solution (Beyotime) for 20 min, and blocked with Immunol staining blocking buffer (Beyotime) for 1 h at room temperature. Then cells were incubated with goat anti-rabbit antibody LC3 (1:50) overnight at 4℃, washed with PBS, and incubated with Alexa Fluor 488-conjugated secondary antibody (1:500, Thermo Fisher Scientific) or Alexa Fluor 594-conjugated secondary antibody (1:500, Thermo Fisher Scientific) for 1 h at room temperature. DAPI was used to stain the nucleus. Fluorescence signals were observed using a fluorescence microscope.

### Gene specific knockdown

As described previously 27, a lentivirus system (GeneChem, Shanghai, China) was used to knock down the expression of PRKAA1 which encodes for AMPKα according to the manufacturer's instructions. Briefly, cells were grown in 6 mm culture dishes at density of 5 × 10^4^ cells per dish and transduced with lentiviral shRNA with 10 multiplicity of infection (MOI) for 12 h. Then cells were cultured in DMEM containing 10% FBS for 72 h. A nonsilencing scrambled EGFP negative matched shRNA is as control. After transfection, the cells were treated with BA as described previously and harvested for further investigations.

### Xenograft tumor model

All animal studies were approved by the Experimental Animal Ethics Committee of School of Pharmacy, Fudan University (Ethical approval number: 2018-03-HS-HJH-01). Male C57BL/6 mice (5-6 weeks old) were obtained from SIPPR/BK Laboratory Animal Ltd. (Shanghai, China). The animals were housed in a temperature-controlled room (22 ± 2 ℃) with a 12 h light-dark cycle and had free access to standard mouse chow and water.

After 7 days' acclimation, 3 × 10^5^ LLC cells suspended in 0.2 mL cold PBS were subcutaneously injected into the right flank of each mouse. When the xenografts reached ~5 mm, the mice were randomly divided into 3 groups and given intragastric administration of 0.2 mL 0.5% CMC-Na (control group) or BA (dissolved in 0.5% CMC-Na, 50 and 100 mg/kg, respectively) every day. Tumor sizes were measured every 2 days by a digital caliper and were calculated by a standard formula: length × width^2^/2. After 11 days' treatment, the animals were sacrificed. Tumor tissues, liver, and kidney were isolated for further study.

### Immunohistochemistry (IHC)

Tumors were excised from each mouse, fixed in 4% paraformaldehyde, and embedded in paraffin. Paraffin sections were processed by means of routine histological techniques and stained with hematoxylin and eosin (H&E). Immunohistochemical staining was carried out manually using antibodies against p-AMPKα, p-Drp1, and Drp1. TUNEL staining was performed according to the manufacturer's instructions. The expression levels were visualized using a fluorescence microscope and a light microscope.

### Statistical analysis

Data were presented as mean ± SEM. Statistical analyses were performed using One-way ANOVA, Two-way ANOVA or unpaired t-tests by GraphPad Prism 5. Differences at p < 0.05 were considered statistically significant.

## Results

### BA inhibited cell viability and induced apoptosis in A549 and H1299 cells

The results showed that BA dose-dependently inhibited viability of A549 and H1299 cells, and H1299 cells were more sensitive to BA than A549 cells (Fig 1b). According to the results of BA-induced cell proliferation inhibition, the concentrations of 80, 120 and 160 μM were used in the subsequent studies. Flow cytometry analysis revealed that BA induced apoptosis in a dose-dependent manner (Fig 1c, d). Apoptosis analysis was also performed by DAPI staining. The results of fluorescence microscopy further revealed that BA induced apoptotic chromatin condensation in a dose-dependent manner in both cell lines (Fig 1e, f). Furthermore, BA promoted the activity of caspase 3 in a dose-dependent manner (Fig 1g). In short, these results suggested that BA induced apoptosis in a dose-dependent manner in A549 and H1299 cells.

### BA activated mitochondrial apoptotic pathway in A549 and H1299 cells

**Figure 2:**
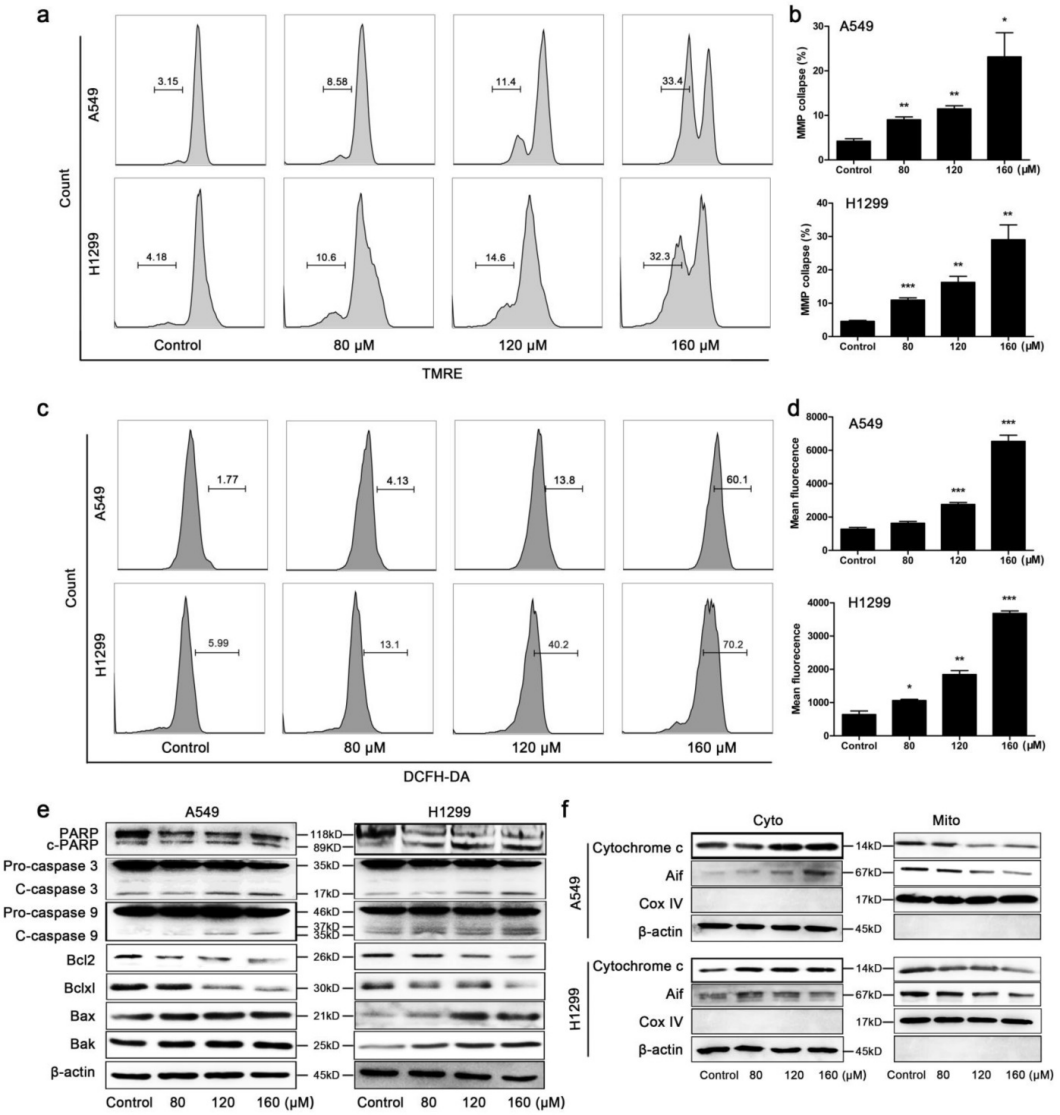
** BA activated mitochondrial apoptotic pathway in A549 and H1299 cells.** (a) Mitochondrial membrane potentials (MMP) collapse in A549 and H1299 cells was monitored by TMRE staining and detected using flow cytometry. (b) MMP collapse was analyzed using FlowJo. (c) Intracellular ROS levels were determined using DCFH-DA staining and detected using flow cytometry. (d) ROS levels were analyzed using FlowJo. (e) The cleavage of PARP, caspase 3 and caspase 9 and protein expression levels of Bcl2 family members (Bcl2, Bclxl, Bax, Bak) were analyzed by western blotting. (f) Cytochrome c and Aif released from the mitochondria (Mito) to the cytoplasm (Cyto) were analyzed by western blotting. Cox IV was used as a loading control for mitochondrial gradient; β-actin was used as a loading control for cytosolic gradient. Data were from at least three independent experiments. *p<0.05, **p<0.01, ***p<0.001

The collapse of MMP was markedly observed in A549 and H1299 cells after BA treatment for 48 h in a concentration-dependent manner (Fig 2a, b). Overproduction of ROS is tightly related to mitochondrial apoptotic pathway. We found that BA treatment significantly increased ROS generation in a dose-dependent manner (Fig 2c, d). Protein expression levels were further investigated. BA treatment increased the cleavage of PARP, caspase 3 and caspase 9 in both A549 and H1299 cells (Fig 2e). BA treatment resulted in a significant decrease of Bcl2 and Bclxl expression levels and increase in Bax and Bak expression levels (Fig 2e). Moreover, BA treatment increased the expression levels of cytochrome c and apoptosis inducing factor (Aif) in cytoplasm and decreased the expression levels of them in mitochondria (Fig 2f).

### BA induced Drp1-mediated mitochondrial fission and mitochondrial impairment in A549 and H1299 cells

**Figure 3:**
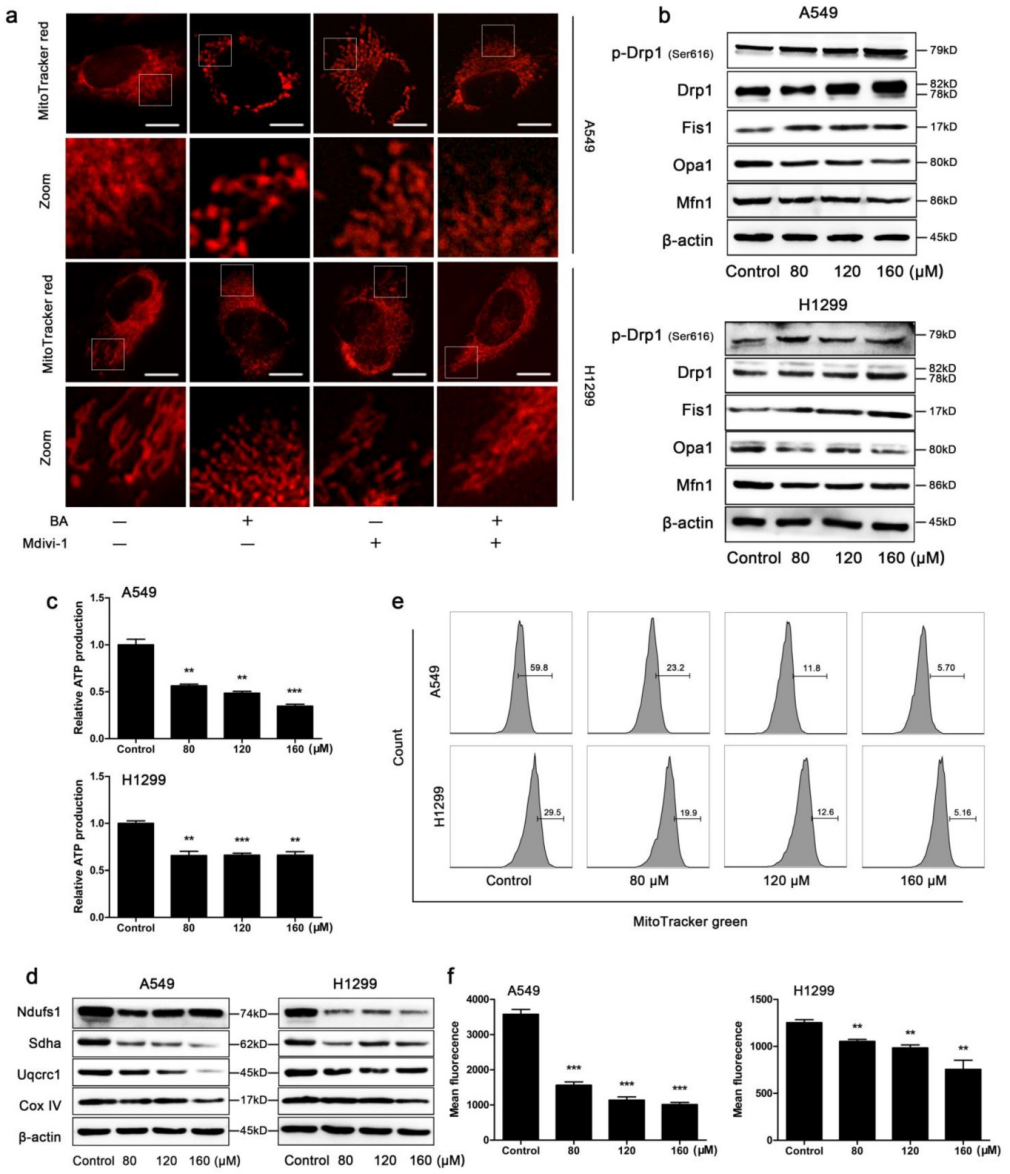
** BA induced Drp1-mediated mitochondrial fission and mitochondrial dysfunction in A549 and H1299 cells.** (a) Cells were pretreated with mdivi-1 (15 μM) for 3 h, followed by treatment with BA (120 μM) for 48 h. Mitochondrial morphology was performed by MitoTracker red staining and detected by fluorescent microscopy (scale bar, 10 μm). (b) The expression levels of mitochondrial fission and fusion (Opa1, Mfn1) related proteins were determined by western blotting. (c) ATP production was determined using an ATP assay kit. (d) The expression levels of mitochondrial respiratory chain complexes were determined by western blotting. (e) Mitochondrial mass was determined by MitoTracker green staining and detected by flow cytometry. (f) Mean fluorescence intensity was analyzed using FlowJo. Data were from at least three independent experiments. **p<0.01, ***p<0.001

When the cells were stained with the mitochondrial-specific fluorescence probe MitoTracker red, mitochondria presented as smaller and punctate structures in BA-treated cells, while mitochondria presented as elongated filamentous structures in control cells (Fig 3a). To further study the role of Drp1, the most important regulator of mitochondrial fission, we used mdivi-1 to selectively inhibit Drp1 and found that mdivi-1 weakened BA-induced mitochondrial fission (Fig 3a). In addition, BA exposure increased the expression levels of mitochondrial fission related proteins such as Drp1, p-Drp1 (Ser616) and Fis1 and decreased the expression levels of mitochondrial fusion related proteins such as Opa1 and Mfn1 (Fig 3b). Furthermore, we observed energy and function alterations upon BA treatment. The results showed that BA significantly reduced ATP production (Fig 3c), the expression levels of mitochondrial respiratory chain complexes (Fig 3d) and mitochondrial mass (Fig 3e, f). Taken together, these findings suggested that BA induced mitochondrial fission and mitochondrial impairment.

### BA induced autophagy and autophagic flux in A549 and H1299 cells

**Figure 4:**
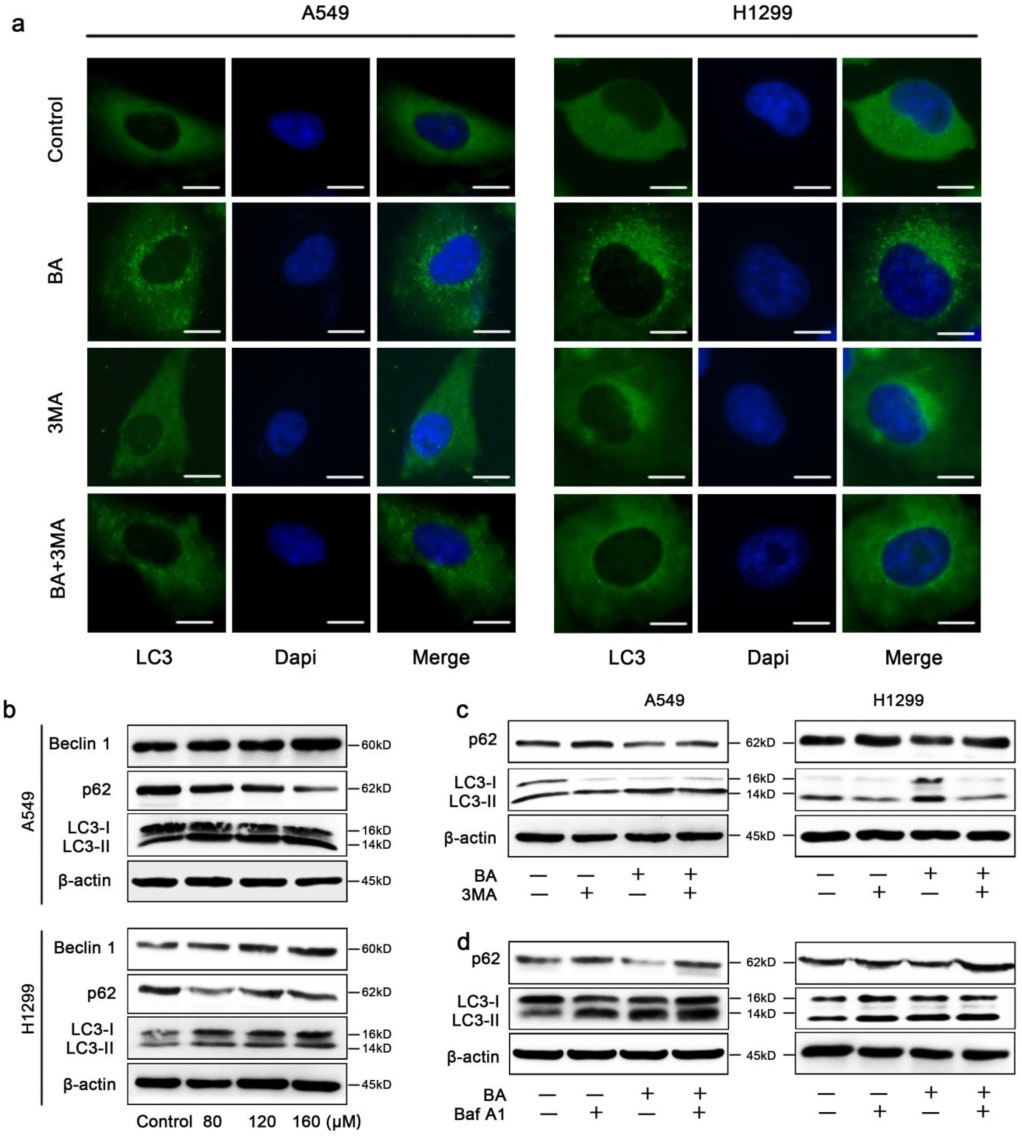
** BA induced autophagy and autophagic flux in A549 and H1299 cells.** (a) A549 and H1299 cells were pretreated with 3-MA (5 mM) for 3 h, followed by treatment with BA (120 μM) for 48 h. LC3 puncta was detected by staining with LC3 antibody and nucleus was detected by staining with DAPI through immunofluorescence using fluorescence microscopy (scale bar, 10 μm). (b) The expression levels of autophagy related proteins were determined by western blotting. (c) A549 and H1299 cells were pretreated with 3-MA (5 mM) for 3 h, followed by treatment with BA (120 μM) for 48 h. The expression levels of autophagy related proteins were determined by western blotting. (d) A549 and H1299 cells were pretreated with Baf-A1 (10 nM) for 3 h, followed by treatment with BA (120 μM) for 48 h. The expression levels of autophagy related proteins were determined by western blotting.

BA significantly increased endogenous LC3 puncta compared to the control group in A549 and H1299 cells (Fig 4a). Meanwhile, western blotting results revealed that BA increased conversion of LC3-I to LC3-II and the expression levels of Beclin 1 (Fig 4b). A complete autophagy process includes autophagic flux. We observed the reduction of the expression levels of p62, an autophagic substrate, in BA-treated cells (Fig 4b). Combinational treatment with 3-MA (an early-stage autophagy inhibitor) reduced endogenous LC3 puncta (Fig 4a). Consistently, BA combined with 3-MA decreased conversion of LC3-I to LC3-II and increased p62 levels (Fig 4c). On the contrary, Baf-A1 (a late-stage autophagy inhibitor) combined with BA increased conversion of LC3-I to LC3-II and levels of p62 (Fig 4d). Above results demonstrated that BA induced autophagy and autophagic flux in lung cancer cells.

### Mdivi-1 weakened BA-induced apoptosis in A549 and H1299 cells

**Figure 5:**
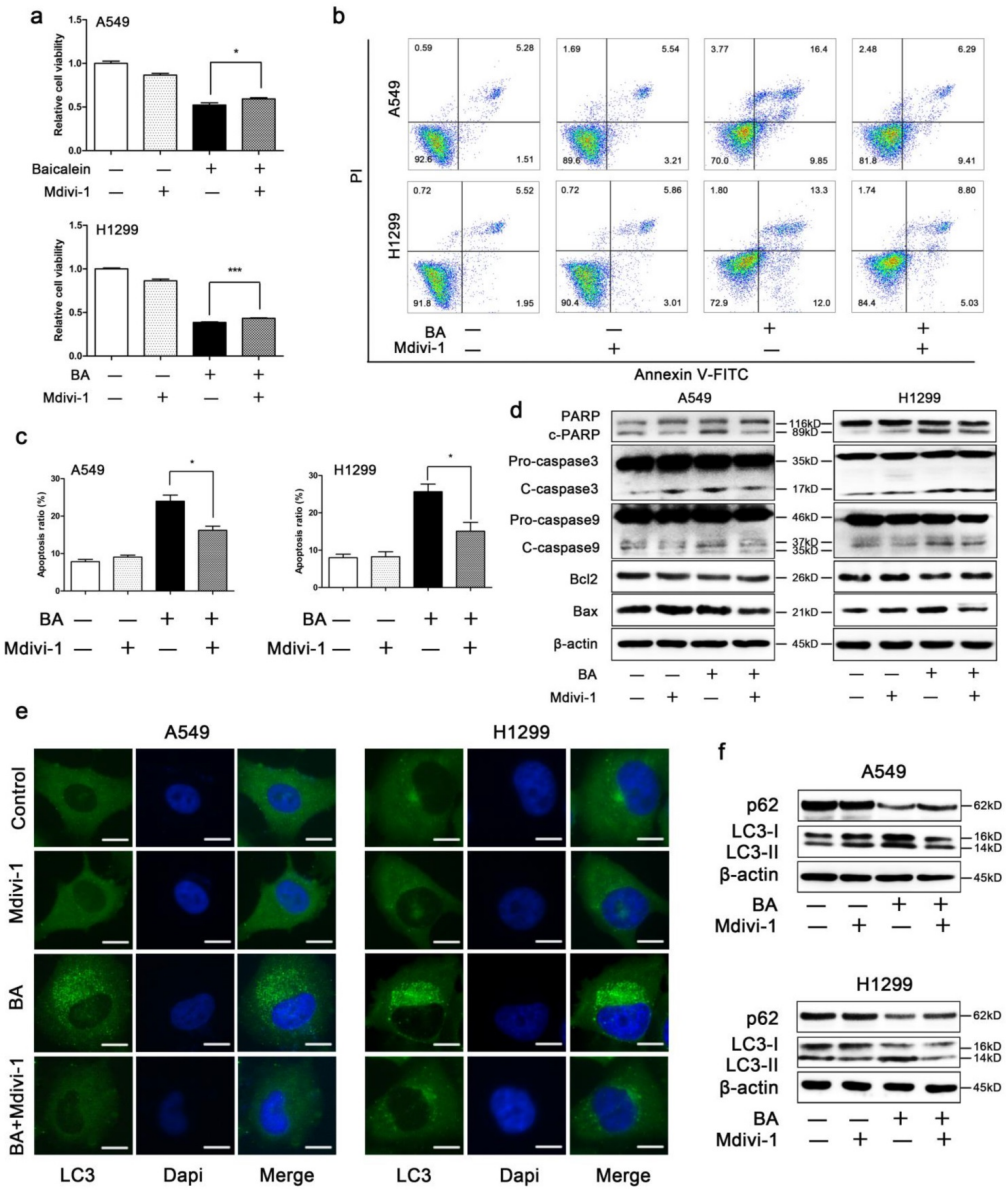
** Mdivi-1 weakened BA-induced apoptosis and autophagy.** A549 and H1299 cells were pretreated with mdivi-1 (15 μM) for 3 h, followed by treatment with BA (120 μM) for 48 h. (a) Cell viability was measured by WST-1 assay. (b) Apoptosis was performed by staining with Annexin V-FITC and PI and detected using flow cytometry. (c) Apoptosis ratio was analyzed using FlowJo. (d) The expression levels of mitochondrial apoptotic pathway related proteins were determined by western blotting. (e) LC3 puncta was detected by immunofluorescence using fluorescence microscopy (scale bar, 10 μm). (f) The expression levels of autophagy related proteins were determined by western blotting. Data were from at least three independent experiments. *p<0.05, ***p<0.001

The results showed that mdivi-1 attenuated BA-induced loss of cell viability (Fig 5a) and BA-induced apoptosis (Fig 5b, c). Western blotting showed that mdivi-1 decreased BA-induced cleavage of PARP, caspase 3 and caspase 9 (Fig 5d). In addition, we also found that mdivi-1 increased the expression levels of Bcl2 and decreased the expression levels of Bax (Fig 5d).

### Mdivi-1 weakened BA-induced autophagy in A549 and H1299 cells

In order to investigate whether mitochondrial fission plays a role in BA-induced autophagy, we treated A549 and H1299 cells with a combination of BA and mdivi-1. We found that mdivi-1 significantly decreased BA-induced endogenous LC3 puncta (Fig 5e). Western blotting showed that mdivi-1 decreased the conversion of LC3-I to LC3-II and increased the expression levels of p62 (Fig 5f).

### BA activated AMPK signaling pathway in A549 and H1299 cells

**Figure 6:**
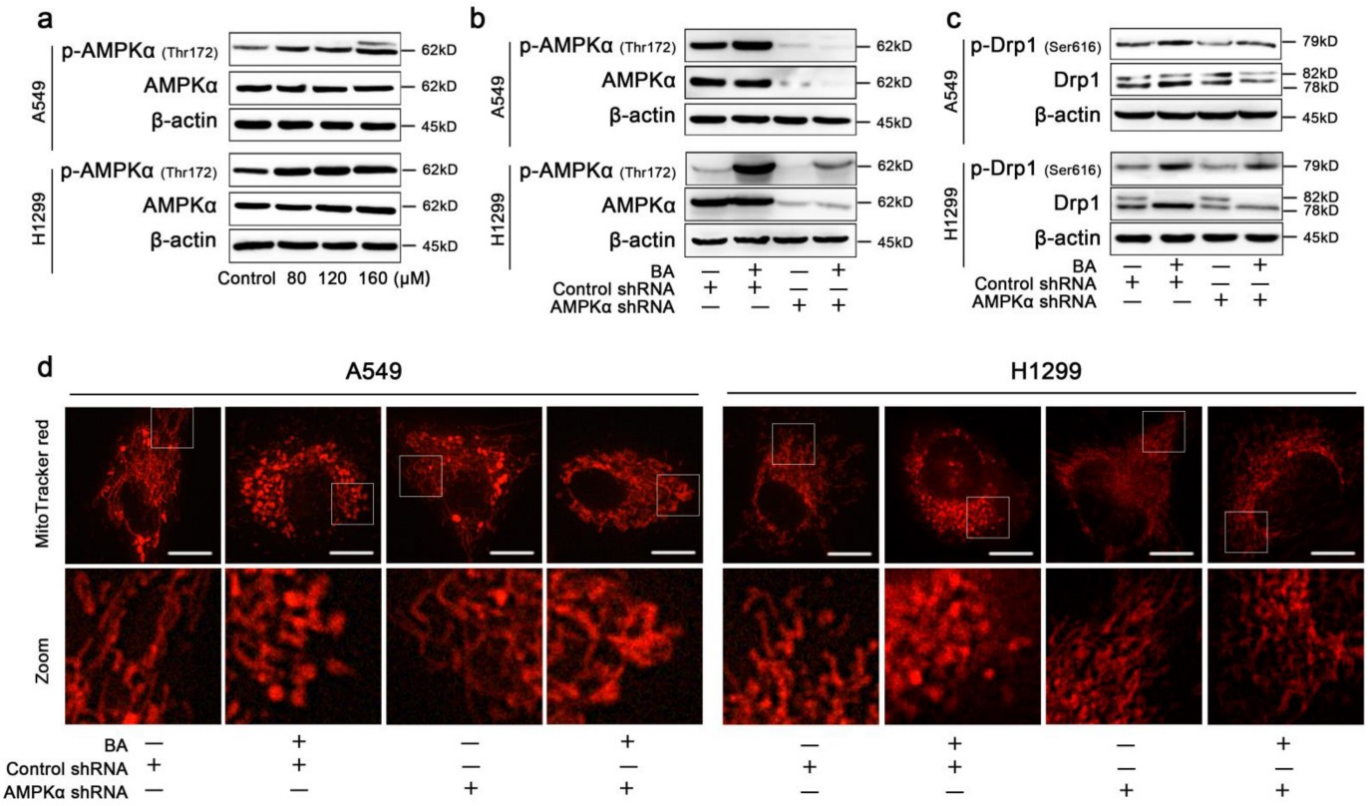
** BA induced Drp1-mediated mitochondrial fission via activation of AMPK pathway.** (a) The expression levels of AMPKα and p-AMPKα (Thr172) were determined by western blotting. (b) A549 and H1299 cells were transfected by AMPKα lentivirus and control lentivirus and treated with or without BA (120 μM) for 48 h. The expression levels of AMPKα and p-AMPKα (Thr172) were determined by western blotting. (c) The expression levels of Drp1 and p-Drp1 (Ser616) were determined by western blotting. (d) Mitochondria morphology was determined MitoTracker red staining and detected by fluorescent microscopy (scale bar, 10 μm).

To additionally examine the mechanism by which BA-induced mitochondrial fission mediated apoptosis and autophagy in lung cancer cells, AMPK signaling pathway was studied. The protein levels of AMPKα, p-AMPKα (Thr172) were detected using western blotting after treatment with BA. The results showed that BA dose-dependently increased the expression levels of p-AMPKα in A549 and H1299 cells (Fig 6a).

### Knockdown of AMPK blocked BA-induced mitochondrial fission in A549 and H1299 cells

To confirm AMPK signaling pathway serves as a master regulator of BA-induced mitochondrial fission, the lentiviral shRNA directed against PRKAA1 was used to block AMPKα. Knockdown of AMPKα blocked the protein expression of AMPKα and p-AMPKα (Fig 6b). Furthermore, western blotting assay showed that knockdown of AMPKα decreased the expression levels of Drp1 and p-Drp1 (Ser616) (Fig 6c). MitoTracker red staining analysis also showed that knockdown of AMPKα increased the length of mitochondria and blocked BA-induced mitochondrial fission (Fig 6d).

### Knockdown of AMPKα attenuated BA-induced apoptosis and autophagy in A549 and H1299 cells

**Figure 7:**
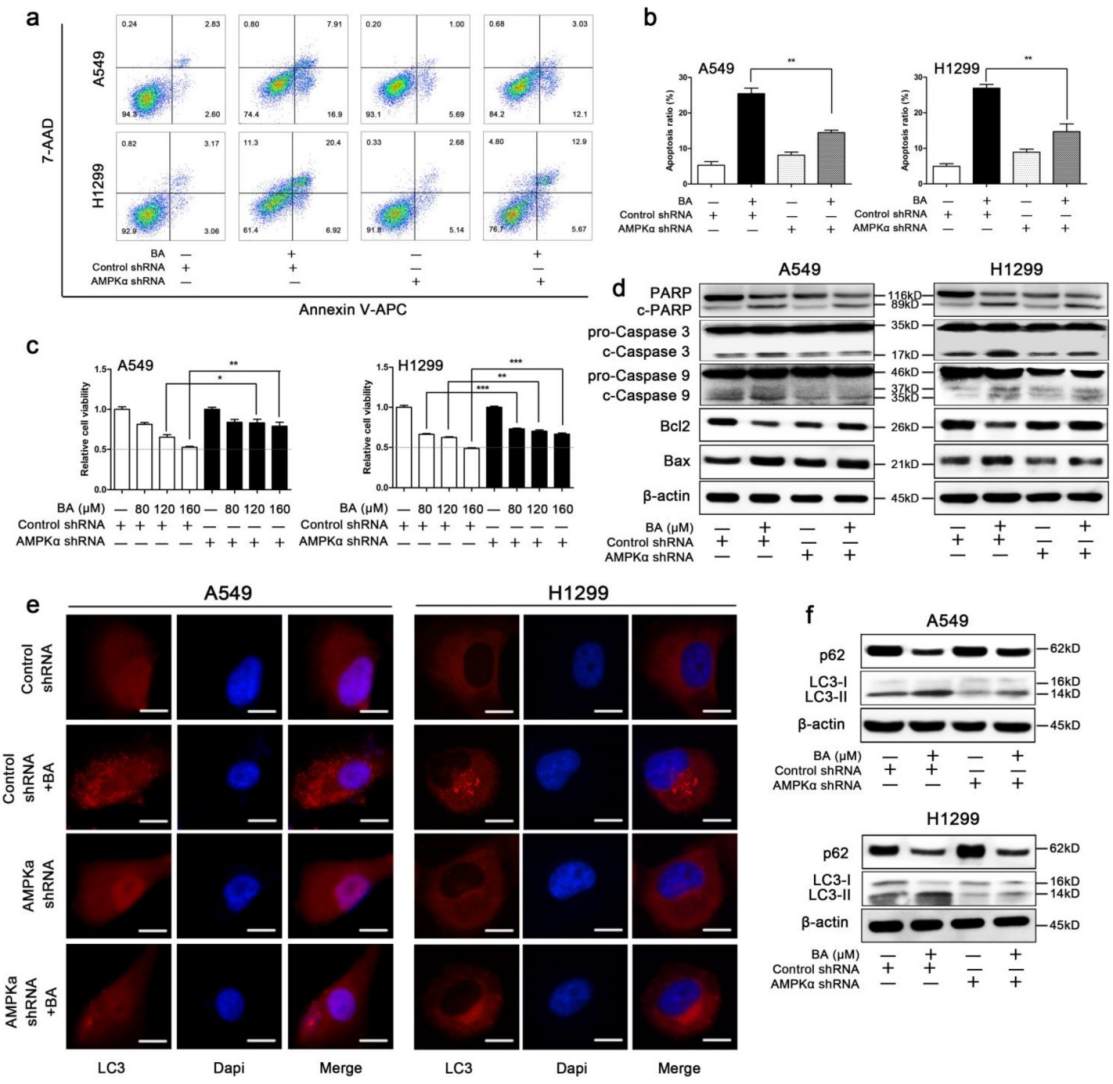
** BA induced apoptosis and autophagy via activation of AMPK pathway.** (a) Apoptosis analyses were performed by staining with Annexin V-APC and 7-AAD and detected using flow cytometry. (b) Apoptosis ratio was analyzed using FlowJo. (c) Cell viability was measured by WST-1 assay. (d) The expression levels of mitochondrial apoptotic pathway related proteins were determined by western blotting. (g) LC3 puncta was detected by immunofluorescence using fluorescence microscopy (scale bar, 10 μm). (f) The expression levels of autophagy related proteins were determined by western blotting. Data were from at least three independent experiments. *p < 0.05, **p < 0.01, ***p < 0.001

We then explored whether AMPK signaling pathway is involved in mitochondrial fission-mediated apoptosis and autophagy. BA induced apoptosis, while knockdown of AMPKα partly abolished this effect (Fig 7a, b). WST-1 assay demonstrated that knockdown of AMPKα abolished BA-induced the decrease of cell viability (Fig 7c). These findings were further supported by western blotting. Knockdown of AMPKα abrogated BA-induced cleavage of PARP, caspase3 and caspase9, increased the expression levels of Bcl2, and decreased the expression levels of Bax (Fig 7d). These above results suggested that knockdown of AMPKα abrogated BA-induced apoptosis.

BA induced endogenous LC3 puncta, while AMPKα downregulation significantly eliminated this effect (Fig 7e). Meanwhile, knockdown of AMPKα decreased BA-induced conversion of LC3-I to LC3-II and increased the expression levels of p62 (Fig 7f). Taken together, these results revealed that knockdown of AMPKα abrogated BA-induced autophagy.

### BA inhibited tumor growth by induction of apoptosis and autophagy via AMPK/Drp1/ mitochondrial fission pathway in LLC xenograft mouse model

**Figure 8:**
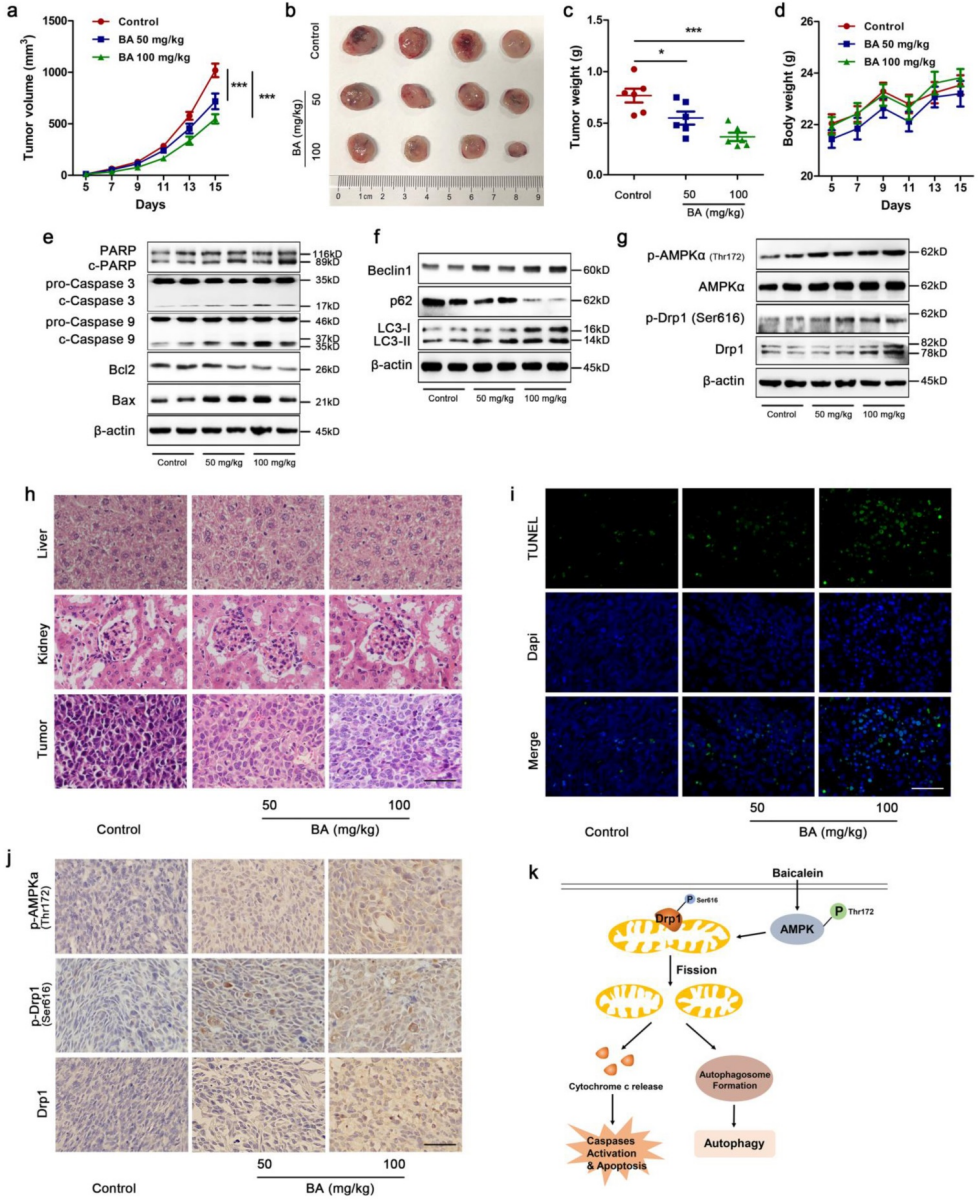
** BA inhibited tumor growth *in vivo* by apoptosis and autophagy via activation of the AMPK/mitochondrial fission pathway.** (a) Tumor volumes in LLC xenograft mice were measured every two days in the control and BA (50 mg/kg and 100 mg/kg) groups (n = 10). (b) Gross images of tumors. (c) Weight of tumors (n = 6). (d) Changes of mice weight in each group over time (n = 10). The expression levels of mitochondrial apoptotic pathway related proteins (e), autophagy related proteins (f), and AMPK pathway related proteins (g) were determined by western blotting. (h) The morphology of mouse liver, heart and tumor was determined by H&E stanning (scale bar, 50 μm). (i) Apoptosis ratio was determined by TUNEL assay (scale bar, 50 μm). (j) The immunoreactivity of p-AMPKα, p-Drp1, and Drp1 were detected by IHC (scale bar, 50 μm). (k) A proposed model for BA-induced apoptosis and autophagy through AMPK-Drp1-mitochondrial fission pathway. *p<0.05, ***p<0.001

To investigate whether BA exhibits anti-tumor activity *in vivo* and the underlying mechanism, a LLC xenograft mouse model was established and received intragastric administration of either vehicle or BA (50 mg/kg and 100 mg/kg) for 11 days. Treatment with BA at doses of 50 and 100 mg/kg both resulted in a significant inhibition of tumor growth compared to the control group (Fig 8a-c). There were no significant changes in body weight among BA-treated mice and control mice (Fig 8d). Western blotting showed that BA at doses of 50 and 100 mg/kg both increased cleavage of PARP, caspase 3 and caspase 9, promoted the protein levels of Bax and decreased the protein levels of Bcl2 (Fig 8e). BA increased the expression levels of Beclin1 and conversion of LC3-I to LC3-II, and decreased the expression levels of p62 (Fig 8f). Meanwhile, BA at doses of 50 and 100 mg/kg both increased the protein levels of p-AMPKα, p-Drp1 and Drp1 (Fig 8g). H&E stanning revealed that BA treatment showed no significant morphological changes in liver and kidney compared to the control group (Fig 8h). Compared to the control group, necrosis and infiltration of inflammatory cells increased in tumor sections of BA-treated mice (Fig 8h). Furthermore, TUNEL assay revealed that BA increased apoptosis ratio (Fig 8i). BA increased the immunoreactivity of p-AMPKα, p-Drp1 and Drp1 (Fig 8j). Taken together, we summarized the above data and proposed a model for BA-induced apoptosis and autophagy through AMPK-Drp1-mitochondrial fission pathway in lung cancer (Fig 8k).

## Discussion

Mitochondria have a unique ability to regulate their morphology in response to various cellular stimuli. Mitochondrial fission, one type of mitochondrial dynamics, is important for various cellular processes such as apoptosis 28,29 and mitochondrial clearance through mitophagy 30. Several studies have demonstrated that cells with mitochondria observed small, round and more numerous organelles morphologically were followed by apoptosis 12,31. Mitochondrial fission clearly depends on Drp1 32. In response to apoptotic stimuli, the decrease of MMP and the release of cytochrome c are mediated by Bax-lined pores at sites of Drp1-mediated mitochondrial fission 33. Inhibition of Drp1 before inducing apoptosis not only inhibits mitochondrial fission but also delays loss of MMP, caspase activation and the process of cell death itself 12. In the present study, we showed that BA increased apoptosis ratio in lung cancer cells. BA increased MMP collapse, ROS production and cleavage of PARP, caspase 3 and caspase 9. BA also increased the release of cytochrome c and Aif from mitochondria to cytoplasm. These results suggested that BA activated mitochondrial apoptotic pathway and induced apoptosis, which is consistent with other studies 18,20,21,34. Moreover, BA increased mitochondrial fragmentation, promoted mitochondrial fission related protein expressions, and impaired mitochondrial functions such as cellular ATP depletion, decrease of mitochondrial mass and decrease of mitochondrial respiratory chain related protein expressions. In addition, autophagy degrades cellular components and can be prevented with a dominant negative mutant of Drp1, suggesting that autophagy requires Drp1 35. Mitochondrial fission facilitates autophagic clearance of dysfunctional mitochondria. Here, we found that BA induced autophagy and activated autophagic flux in lung cancer cells. Then after inhibiting mitochondrial fission with mdivi-1, BA-induced apoptosis and autophagy were partly abolished. The above results were also supported by *in vivo* data. BA increased the expression levels of Drp1 and p-Drp1 (Ser616) by western blotting and IHC. Therefore, our data suggested that Drp1-mediated mitochondrial fission may play an essential role in BA-induced apoptosis and autophagy in lung cancer.

Drp1-mediated mitochondrial fission is regulated by a series of post-translational modifications such as phosphorylation 9,35, ubiquitination 37 and sumoylation 38. Phosphorylation has been reported to control Drp1's activity. Phosphorylation at Ser616 facilitates mitochondrial fission, leading to apoptosis 39-41. AMPK, a master sensor of energy stress, can be activated (mainly phosphorylation at Thr172) by a wide variety of mitochondrial stimuli and acutely triggers mitochondrial fission 17,41. Previous studies also found that activation of AMPK inhibited cell proliferation, promoted apoptosis 16 and induced autophagy 17. Therefore, AMPK is an essential target for cancer therapy. Our data showed that BA activated AMPK pathway through phosphorylation at Thr172 in lung cancer cells. Knockdown of AMPKα using a lentivirus system blocked BA-induced phosphorylation of Drp1 and Drp1-mediated mitochondrial fission. Moreover, knockdown of AMPKα blocked BA-induced apoptosis and autophagy. *In vivo*, BA increased the expression levels of p-AMPK (Thr172) detected by western blotting and IHC. These results suggested that BA-induced mitochondrial fission mediated apoptosis and autophagy were regulated by AMPK pathway. Several studies revealed that BA indeed activates AMPK pathway, contributing to autophagy and/or apoptosis 42,43. However, our study established the link between Drp1-mediated mitochondrial fission and autophagy/apoptosis induced by BA.

Previous studies have found that BA induces apoptosis and autophagy in multiple cancer cells 18,20,21,26, but the underlying mechanisms are still unclear. In the present study, we demonstrated that BA induced apoptosis and autophagy via activating Drp1-mediated mitochondrial fission in lung cancer A549 and H1299 cells. Subsequently, we showed that AMPK signaling pathway contributed to Drp1-mediated mitochondrial fission. Blocking AMPK pathway attenuated BA-induced mitochondrial fission, apoptosis and autophagy. These results were further clarified *in vivo*. BA inhibited tumor growth in a LLC xenograft mouse model. Meanwhile, BA induced apoptosis, autophagy and mitochondrial fission *in vivo*. AMPK pathway was activated as well by BA treatment. Therefore, our data revealed the role of AMPK/Drp1/mitochondrial fission axis in regulating BA-induced apoptosis and autophagy in lung cancer.

## Conclusions

In conclusion, our study demonstrated that BA, a natural compound, exhibited anticancer activities against A549 and H1299 lung cancer cells *in vitro* and LLC xenograft *in vivo*. BA activated AMPK pathway and enhanced fatal Drp1-mediated mitochondrial fission. Excessive mitochondrial fission caused mitochondrial dysfunction, apoptosis and autophagy. Our data revealed a new pathway by which BA exerts anti-lung cancer efficacy.
